# Endocrinopathies in Thalassemia major patients in Thalassemia Center Jakarta, Indonesia

**DOI:** 10.1186/1687-9856-2013-S1-P58

**Published:** 2013-10-03

**Authors:** Frida Soesanti, Siti Ayu Putriasih, Aman Pulungan, Pustika Amalia Wahidiyat

**Affiliations:** 1 Pediatric Endocrinology Division, Universitas Indonesia-Cipto Mangukusumo Hospital, Jakarta, Indonesia; 2 Registrar in Thalassemia Center; 3 Hemato-Oncology Division, Universitas Indonesia-Cipto Mangunkusumo Hospital, Jakarta, Indonesia

## Background

Regular transfusion in thalassemia major patients increases life expectancy and improves quality of life, but results in iron overload, which had toxic effects to organs including endocrine glands. The introduction of iron chelation therapy has reduced its toxicity, but complications may still occur. In Indonesia, most of our patients did not receive optimal iron chelation therapy, which might result in higher prevalence of endocrine complications.

## Aims

To find out the endocrinopathies profile in thalassemia major patients in Thalassemia Center Jakarta.

## Methods

This was a retrospective study based on the registry database in Thalassemia Center, Jakarta. We included patients who diagnosed as thalassemia major with complete data on glucose metabolism, thyroid function, pituitary-gonadal axis, bone profile, bone age, and serum ferritin level. We analyzed the association between ferritin level, chelation therapy and type of thalassemia with endocrine profile using chi-square with the significant value of 0.05.

## Results

Complete data on endocrine profile were found in 67 subjects (31 boys, 36 girls), 23 (34%) were diagnosed as β-thalassemia homozygote and the rest as β-thalassemia/HbE. The mean age was 16.7±5.8 years. Most of the patients (63%) received iron chelation therapy with desferrioxamine followed by deferiprone (20%) and only two patients have not taken any iron chelation therapy yet. Short stature was found in 65% of subjects, while 20% of subjects suffered from delayed puberty, 41% had hypothyroidism, and 29% had retarded bone age. None of them was diagnosed as DM or IGT, but one diagnosed as IFG. Hypocalcemia was found in 27% subjects. Subjects with serum ferritin level ≥ 2,500 ng/mL had increased risk to develop hypothyroidism, hypocalcemia and hyperphosphatemia, even though not statistically significant (p=0.58, p=0.08, respectively). Serum ferritin level also not associated with short stature and delayed puberty. Subjects with thalassemia beta-major had increased risk to develop hypothyroidism (p=0.036), but no differences found in the prevalence of short stature, delayed puberty, hypocalcemia, and hypophosphatemia (p0.17, p=0.91, p=0.60, respectively). The frequency of transfusion per year and type of chelation therapy did not influence the endocrine profiles.

## Conclusions

This study showed that the prevalence of short stature among thalassemia patients is higher in Thalassemia Center Jakarta, while the risk to develop impaired glucose metabolism is lower, despite of poor compliance in iron chelation therapy. The risk to develop hypothyroidism, delayed puberty, and hypoparathyroidism were comparable to other studies.

